# ASCL1 phosphorylation and *ID2* upregulation are roadblocks to glioblastoma stem cell differentiation

**DOI:** 10.1038/s41598-022-06248-x

**Published:** 2022-02-11

**Authors:** Roberta Azzarelli, Aoibheann McNally, Claudia Dell’Amico, Marco Onorati, Benjamin Simons, Anna Philpott

**Affiliations:** 1 Wellcome - Medical Research Council Cambridge Stem Cell Institute, University of Cambridge, Cambridge, UK; 2 Department of Oncology, Hutchison-MRC Centre, Hills Road, Cambridge, CB2 0XZ UK; 3 Department of Biology, Unit of Cell and Developmental Biology, University of Pisa, Pisa, Italy; 4 The Wellcome Trust/Cancer Research UK Gurdon Institute, University of Cambridge, Cambridge, UK; 5 Department of Mathematics, University of Cambridge, Cambridge, UK

**Keywords:** Cancer, Cell biology, Developmental biology, Stem cells

## Abstract

The growth of glioblastoma (GBM), one of the deadliest adult cancers, is fuelled by a subpopulation of stem/progenitor cells, which are thought to be the source of resistance and relapse after treatment. Re-engagement of a latent capacity of these cells to re-enter a trajectory resulting in cell differentiation is a potential new therapeutic approach for this devastating disease. ASCL1, a proneural transcription factor, plays a key role in normal brain development and is also expressed in a subset of GBM cells, but fails to engage a full differentiation programme in this context. Here, we investigated the barriers to ASCL1-driven differentiation in GBM stem cells. We see that ASCL1 is highly phosphorylated in GBM stem cells where its expression is compatible with cell proliferation. However, overexpression of a form of ASCL1 that cannot be phosphorylated on Serine–Proline sites drives GBM cells down a neuronal lineage and out of cell cycle more efficiently than its wild-type counterpart, an effect further enhanced by deletion of the inhibitor of differentiation ID2, indicating mechanisms to reverse the block to GBM cell differentiation.

## Introduction

Glioblastoma (GBM) is the most common malignant brain tumor in adults and, despite advances in research and in treatment modalities, prognosis is still poor, with overall 5-year survival being less than 5%^1–3^. A common feature of this class of aggressive tumors is recurrence after treatment, accompanied by resistance to further therapies. Research in the past two decades has identified and characterized the presence of stem cells within these tumors^4,5^ that are capable not only of sustaining tumor growth but also of driving relapse after treatment^6,7^; their quiescent nature renders them resistant to antiproliferative agents and allows them to remain after treatment as a reservoir of cells that drive relapse. It has thus become crucial to find ways to target the GBM stem cell compartment and reduce their tumorigenic potential.

GBMs are highly heterogeneous tumors and have been classified into 3 main subgroups based on bulk gene expression studies: the proneural (TCGA-PN), the classical (TCGA-CL) and the mesenchymal (TCGA-MES) clusters^8^. This classification is based on the dominant transcriptional pattern present in tissue at the location of resection. However, more than one subgroup might coexist within a single tumor, as confirmed by single cell sequencing (scRNAseq) of the primary tumor transcriptome^9–11^. These studies also identified the presence of four cellular states, whereby each state is associated with the gene signature of a developmental precursor (astrocyte AC-like, oligodendrocyte precursor OPC-like or neural progenitor NPC-like) or of mesenchymal cells^10^. Given the resemblance of these cellular states to developmental intermediates, it is thus not surprising that developmental factors that regulate proliferation and differentiation of precursors in the embryonic brain are re-expressed in GBM^12^, especially in the PN subtype that contains a prevalence of OPC-like and NPC-like cellular states.

One of these factors is the transcriptional regulator ASCL1, which belongs to the bHLH proneural family of proteins regulating neuronal differentiation in the embryo^13–17^. ASCL1 has been found expressed in a subset of GBMs of the PN subtype^18–21^ and while it is a well-known driver of neuronal differentiation in the embryonic brain, in GBM, it fails to engage a full differentiation programme. Here, we asked what would restrain ASCL1 proneural activity in GBM and explored the possibility that the oncogenic cell cycle environment may modulate ASCL1 phosphorylation, which in turn regulates its expression and activity.

The oncogenic environments of high grade GBM and lower grade gliomas are enriched for active Serine–Proline-directed kinases, such as CDKs^22,23^, which would maintain ASCL1 in a highly phosphorylated form in GBM cells. By comparing the activity of wild type (WT) and of a phospho-mutant form of ASCL1 that cannot be phosphorylated on SP consensus sites, we show that inhibition of ASCL1 phosphorylation increases the fraction of cells expressing neuronal markers and drives GBM cells to a post-mitotic neuronal-like state that is refractory to cell cycle re-entry upon growth factor stimulation. While mitotic exit is not readily reversible under these conditions, the differentiated neuronal-like cells nevertheless fail to exhibit mature characteristics, such as elaborated morphology and mature marker expression. Investigating the potential roadblocks to phospho-mutant ASCL1-mediated differentiation, we reveal an important role for *ID2* in restraining GBM differentiation. Altogether, we propose that manipulation of ASCL1 phosphorylation could be targeted to develop novel therapeutic opportunities for GBM treatment aimed at reducing tumorigenicity and enhancing differentiation.

## Results

### Phosphorylated ASCL1 is expressed in a subset of GBM stem cells

**Figure 1:**
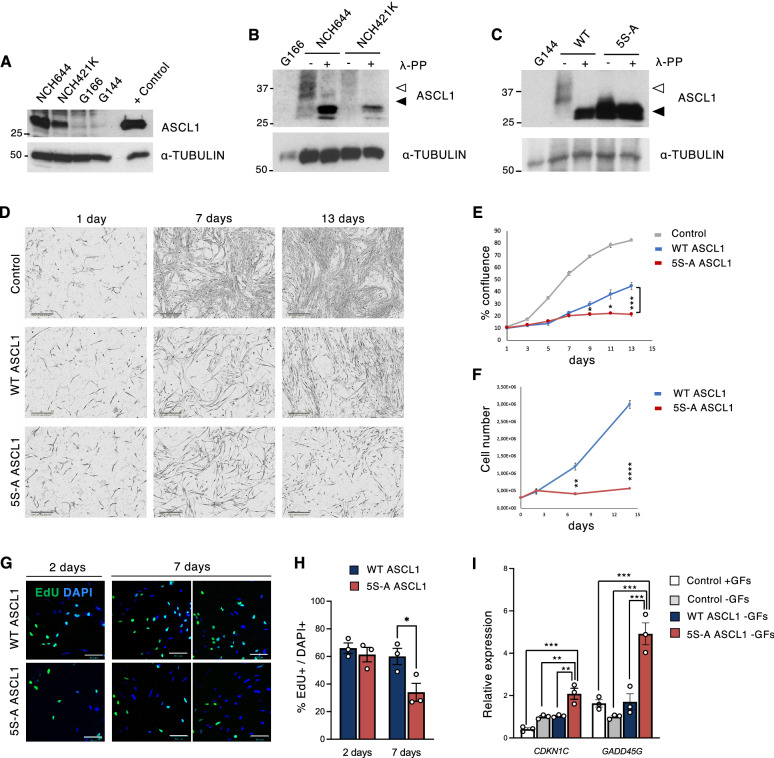
ASCL1 phosphorylation regulates proliferation of human GSCs. (**A**) ASCL1 protein expression in GBM cell lines; positive control is after ASCL1 overexpression. (**B**) Western blot showing endogenous ASCL1 phosphorylation in GBM cells, with and without phosphatase (λ-PP) treatment. (**C**) Overexpression of WT and 5S-A ASCL1 in G144 cells, with and without λ-phosphatase treatment. White arrowheads in **B** and **C** indicate phosphorylated ASCL1, while black arrowheads indicate unphosphorylated ASCL1. (**D**) Representative images of G144 cells after growth factor withdrawal and dox-induced WT and 5S-A ASCL1 expression. Scale bars: 300 μm. (**E**) Quantification of cell confluence. Each data point is mean ± SEM. n = 3 independent experiments; one-way ANOVA followed by the Bonferroni post-hoc test; *p < 0.05; ***p < 0.001. All data points from day 3 onwards statistically differ between control and WT ASCL1 and between control and 5S-A ASCL1 (*p* < 0.05; not shown). (**F**) Quantification of cell number at 2, 7 and 14 days of ASCL1 induction. Data: mean ± SEM n = 3 independent experiments, t-test; **p < 0.01; ****p < 0.0001 (**G**) Immunofluorescence showing EdU incorporation. Scale bars: 100 μm. (**H**) Quantification of the % of EdU-positive cells. Data: mean ± SEM n = 3 independent experiments, t-test; *p < 0.05. (**I**) Expression of negative cell cycle regulators in G144 cells after 7 days of ASCL1 induction. Data: mean ± SEM, normalized to *TBP*. n = 3 independent experiments; one-way ANOVA followed by the Bonferroni post-hoc test; **p < 0.01; ***p < 0.001. Full length western blots are provided in Supplementary Fig. S1A, B and C.

To investigate the potential role of ASCL1 phospho-regulation in glioma stem cells (GSCs), we first sought to determine ASCL1 expression in 4 cell lines derived from primary GBM tumors (Fig. 1A and Supplementary S1; Fig. S2A,B). These cell lines are enriched for glioma stem cells and are grown in the presence of the growth factors EGF and FGF2^24^. In two of the 4 cell lines, ASCL1 protein (Fig. 1A) and ASCL1 transcripts (Fig. S2A) were detected, although mRNA levels were lower than in a positive control SH SY5Y neuroblastoma cell line^25^ (Fig. S2A). Consistent with mRNA expression levels, immunofluorescent staining also showed widespread ASCL1 expression in NCH644 cells, while ASCL1 was not detectable in G144 cells (Fig. S2B). We assessed the phosphorylation status of ASCL1 in the GSC lines by western blot detection of ASCL1 with and without phosphatase treatment, and separation of phosphorylated and un(der)phosphorylated forms of ASCL1 on “phos-tag” gels, which provide better separation of phospho-forms of the protein than standard SDS-PAGE (Fig. 1B). Endogenously expressed ASCL1 runs as a broad slowly migrating band after Phos-tag gel separation, while a more rapidly migrating band appears after phosphatase treatment (Fig. 1B) demonstrating phosphorylation.

### ASCL1 phosphorylation regulates GBM cell proliferation

ASCL1 expression has been associated both with GBM cancer stem-ness^22,26,27^ and progenitor proliferation^14^ and, alternatively, with a more favourable prognosis and a propensity of GBM cells to differentiate upon inhibition of Notch signaling^18^.

We thus tested the effect of over-expression of ASCL1 in the cell line G144 that does not express endogenous ASCL1 (Fig. 1, Fig. S2). As we previously showed that proneural bHLH transcription factors can be highly phosphorylated on multiple SP sites by CDKs (and potentially other SP-targeted kinases), and that multi-site phosphorylation restrains their pro-differentiation activity^28,29^, we set out to test the effect of both wild type (WT) ASCL1 and a mutant version where all 5 SP sites are mutated to Alanine-Proline, 5S-A ASCL1 (Fig. S2C,D).

We generated G144 cells that can inducibly express WT-ASCL1 or phospho-mutant ASCL1 (Fig. S2E,F), by lentiviral transduction of a Tet-ON two-vector doxycycline (dox)-inducible system. Comparing G144 cells transduced with dox-inducible WT and 5S-A *ASCL1* lentiviral vectors using 3 different viral concentrations, we noted that, even when mRNA levels were comparable, 5S-A ASCL1 protein accumulated to significantly higher levels than the wild-type protein, consistent with an increased half-life previously observed for other phospho-mutant bHLHs^25,30^ (Fig. S2E–F). Pools of infected cells expressing similar levels of WT and 5S-A *ASCL1* mRNA expression were chosen for subsequent experiments.

We first checked the phospho-status of overexpressed ASCL1 in G144 cells. Overexpressed WT ASCL1 is highly phosphorylated in G144 cells, as determined by phosphatase treatment (Fig. 1C), and migrates on “phos-tag” gels as a broad slowly migrating band (Fig. 1C, white arrowhead) indicating similar phosphorylation to endogenous ASCL1 in NCH644 and NCH421K cells (Fig. 1B). Induction of WT ASCL1 led to markedly reduced proliferation (Fig. 1D–H), demonstrating that GBM cells that do not normally express ASCL1 can nevertheless respond to its reintroduction by undergoing cell cycle exit.

To determine whether preventing phosphorylation of ASCL1 results in more effective re-engagement of a programme of cell cycle exit, we compared the effect of WT ASCL1 with the mutant 5S-A ASCL1 and found that cells expressing 5S-A ASCL1 grow significantly slower than both parental G144 cells and WT ASCL1-expressing cells (Fig. 1D–F). Interestingly, while monitoring over 13 days, WT ASCL1-expressing cells show a gradual increase in confluency that is not seen after 5S-A ASCL1 over-expression (Fig. 1E), indicating that WT ASCL1 expression can slow the cell cycle but only 5S-A ASCL1 can arrest cell proliferation. Further quantification also shows that WT ASCL1-expressing cells increase in number, while 5S-A-expressing cells do not (Fig. 1F). Moreover, when directly measuring cell proliferation by EdU incorporation at 2 days and 7 days, we saw that, in agreement with cell counts, EdU incorporation was significantly lower after 7 days when cells express phospho-mutant ASCL1 compared to the WT protein (Fig. 1G–H). A similar phenotype was observed when comparing WT and 5S-A induction in the G166 cell line that also does not express endogenous ASCL1 (Fig. S3A–D).

### Regulation of cell cycle by ASCL1

To identify early responses to ASCL1 that could be associated with cell cycle exit, we looked at changes in several positive and negative cell cycle regulators at 24 h after ASCL1 induction (Fig. S4A–B). The expression of most cyclins was decreased or largely unaffected, except for *CCND3* which was upregulated by both forms of ASCL1 (Fig. S4A). CCND3 is often associated with differentiation rather than proliferation during neurogenesis^31^. Focussing on cell cycle inhibitors, *CDKN2A* and *CDKN1B* levels decreased, while *CDKN1A* and *CDKN1C* were upregulated (Fig. S4B). CDKN1A and CDKN1C are known to play important roles in neurogenesis in coordination with bHLH transcription factors^32–36^ and may mediate the cell cycle exit phenotype in GBM. Consistently, we also see upregulation of the CDKN1C protein at different time points after WT and 5S-A ASCL1 induction (Fig. S4C–D) and we see decreased expression and de-phosphorylation of the retinoblastoma protein as soon as 24 h post WT and 5S-A ASCL1 activation (Fig. S4E), also indicating that cells have started to exit cell cycle.

Greater upregulation of *CDKN1C* and of the cell cycle arrest gene *GADD45G* by 5S-A ASCL1 in comparison to WT ASCL1 was observed (Fig. 1I, Fig. S4C,D), and a similar trend in *CDKN1C* activation with 5S-A ASCL1 is seen in the G166 cells (Fig. S4F). Altogether, these results indicate that 5S-A ASCL1 leads to enhanced cell cycle exit. This may be due, in part, to driving more efficient activation of negative cell cycle regulators such as *CDKN1C* and *GADD45G* compared to WT ASCL1.

### 5S-A ASCL1 expressing cells undergo efficient neuronal differentiation

**Figure 2:**
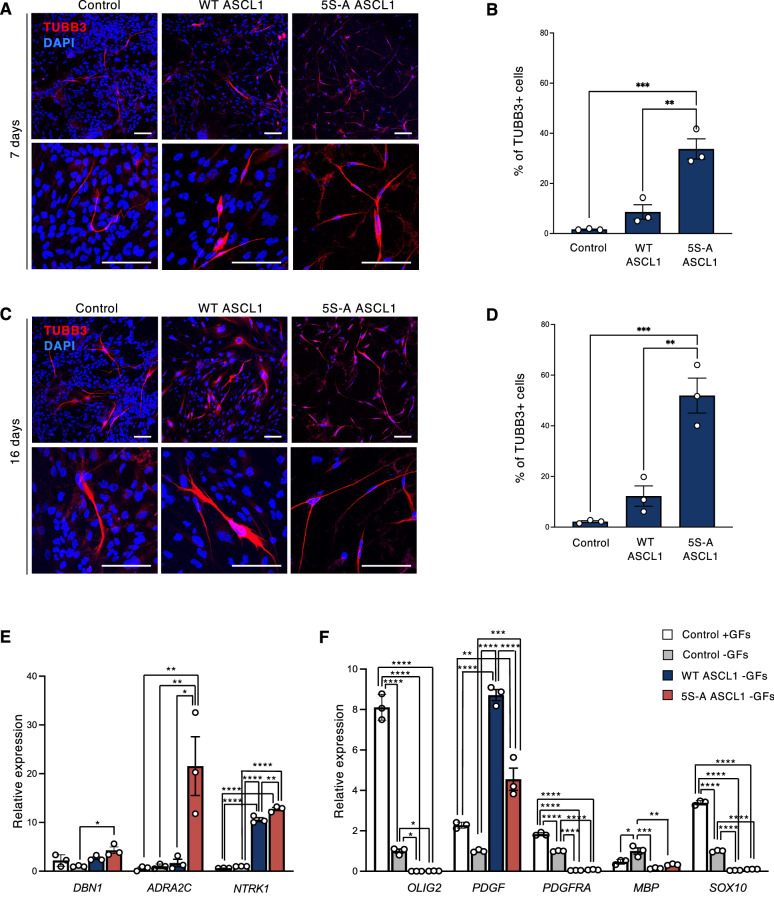
Dephosphorylated ASCL1 promotes neuronal differentiation. (**A**–**C**) Immunostaining for the neuronal marker TUBB3 (red) and quantification of the % of TUBB3^+^ cells over the total DAPI^+^ cells cultured for 7 (**A**,**B**) or 16 days (**C**,**D**) without growth factors and in the presence of dox to induce ASCL1 expression. Scale bars: 100 μm. Data: mean ± SEM n = 3 independent experiments; one-way ANOVA followed by the Bonferroni post-hoc test; **p < 0.01; ***p < 0.001. (**E**,**F**) Relative mRNA expression of different neuronal (**E**) and oligodendrocyte (**F**) markers at 7 days. Data: mean ± SEM, normalized to *TBP*. n = 3 independent experiments; one-way ANOVA followed by the Bonferroni post-hoc test; *p < 0.05; **p < 0.01; ***p < 0.001; ****p < 0.0001.

We then investigated whether the strong proliferative impairment induced by 5S-A ASCL1 expression was coupled to increased neuronal differentiation by looking at the expression of neuronal markers at 7 and 16 days of differentiation. To stimulate GBM cell differentiation we accompanied dox treatment to activate ASCL1 with the withdrawal of the growth factors EGF and FGF2 from the culture media^24^. Immunostaining for TUBB3 and quantification of the fraction of TUBB3^+^ cells showed that only a minor fraction of control and WT ASCL1-overexpressing cells express the neuronal tubulin TUBB3 at both time points (1.6 ± 0.2% of control cells and 8.6 ± 2.9% of WT ASCL1 cells at 7 days; 2.2 ± 0.4% of control cells and 12.2 ± 4.0% of WT ASCL1 cells at 16 days). In contrast, 33.8 ± 4.0% and 52.0 ± 7.0% of 5S-A ASCL1 expressing cells were TUBB3^+^ at 7 and 16 days, respectively (Fig. 2A–D). Enhanced differentiation also accompanied cell cycle exit upon 5S-A ASCL1 expression in the G166 cell line (Fig. S5A–C), indicating that this phenotype is reproducible across cell lines, despite the fact that, unlike G144 cells, G166 cells do not belong to the proneural class and are generally more resistant to differentiation^24^.

We also looked at the expression of other neuronal genes that have been identified as direct targets of ASCL1 in neuroblastoma cells by ChIP-seq and RNA-seq analysis and that are involved in neuro-transmitter release, neuronal growth and sensory neuron specification^29,37–39^. qPCR revealed an increase in expression of *NTRK1* and *ADRA2C* in response to 5S-A ASCL1 expression in comparison to control or WT ASCL1 expressing cells (Fig. 2E), as well as a tendency towards increased expression of *DBN1* (Fig. 2E).

Since G144 cells exhibit an OPC-like phenotype even before differentiation^24^, we investigated whether ASCL1 could also induce oligodendrocyte differentiation in this context. Interestingly, both WT and 5S-A ASCL1 suppressed the expression of oligodendrocyte makers, with the exception of *PDGF* (Fig. 2F). Thus, even in a GSC line predisposed to oligodendrocyte differentiation, ASCL1 does not direct an oligodendrocyte cell fate, but rather drives neuronal differentiation.

### Reversibility and stability of differentiation

**Figure 3:**
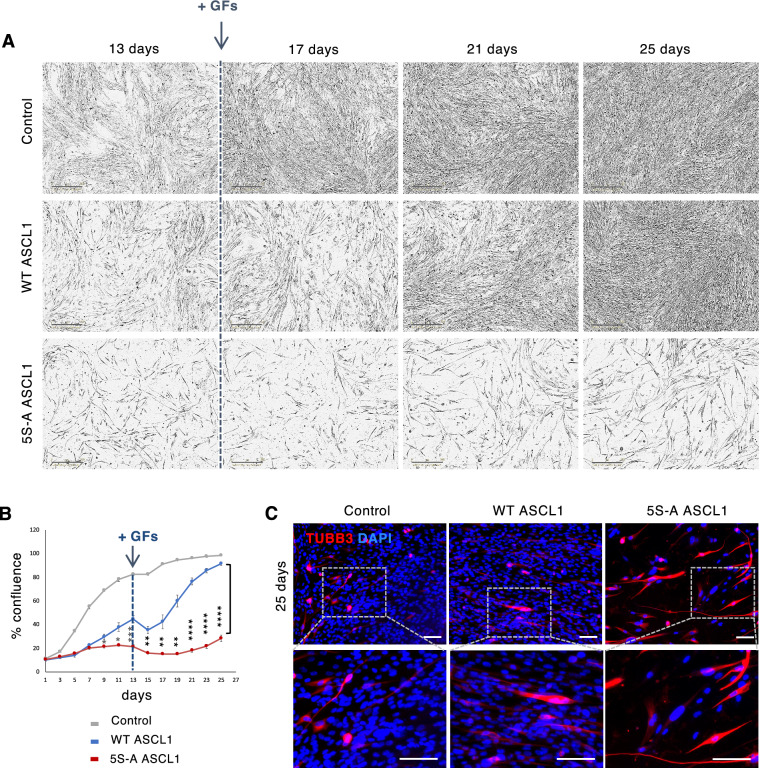
Dephosphorylated ASCL1 promotes stable mitotic exit and differentiation. (**A**) Representative images of G144 cells after removing ASCL1 induction and re-addition of growth factors. Scale bars: 300 μm. (**B**) Quantification of cell confluence. Data: mean ± SEM n = 2 independent experiments for Control + GFs and n = 3 independent experiments for the other conditions; one-way ANOVA followed by the Bonferroni post-hoc test; *p < 0.05; **p < 0.01; ***p < 0.001; ****p < 0.0001. All data points from day 3 onwards are statistically different between control and WT ASCL1 and between control and 5S-A ASCL1 (*p* < 0.05; not shown). (**C**) Immunostaining showing persistent expression of the neuronal marker TUBB3 (red) at 25 days of culture. Scale bars: 50 μm.

Differentiation of GSCs into postmitotic neurons is a potential approach for GBM treatment, but for this to be a viable therapeutic strategy, cells have to remain stably post-mitotic. Previous approaches with this aim have resulted in cell cycle exit that is readily reversed when treatment was discontinued^40^. We next asked whether the strong cell cycle exit seen in response to 5S-A ASCL1 expression was reversed when ASCL1 expression was turned off. After 13 days in differentiation conditions including growth factor withdrawal, we returned cells to growth conditions, adding back the growth factors EGF and FGF2 and removing dox-inducible ASCL1 overexpression (Fig. 3). We found that both control cells and cells that had previously been expressing WT ASCL1 for 13 days start to proliferate between 2 and 4 days after growth factors are added back, quickly reaching full confluency in the subsequent 12 days (Fig. 3A,B). In contrast, phospho-mutant ASCL1-expressing cells remained significantly growth-inhibited, reaching a maximum confluence of only 28.31 ± 2.82% at day 25 (Fig. 3A,B). When stained for TUBB3, a number of 5S-A ASCL1-expressing cells still express the neuronal marker and exhibit elongated morphology characterized by long processes even at day 25, indicating a generally stable differentiated phenotype (Fig. 3C). Taken together, these data demonstrate that cells that have expressed 5S-A ASCL1 for 13 days are more resistant to cell cycle re-entry upon re-exposure to growth factors than cells that have expressed WT ASCL1, and indicate that un(der)phosphorylated ASCL1 can impose a durable mitotic exit.

### *ID2* restrains full 5S-A ASCL1-mediated differentiation

Since 5S-A ASCL1 expression forces GSCs out of cell cycle and towards a TUBB3 + neuronal lineage, but does not seem to drive a mature neuronal phenotype characterized by an elaborated neuronal morphology, we investigated possible interference with 5S-A ASCL1 pro-differentiation activity. BHLH transcription factors usually work in dimeric complexes with ubiquitous partners E proteins, and this dimerization is antagonized by ID proteins that contain the HLH dimerising domain but lack the basic DNA binding domain^41–44^. We hypothesised that, as ID proteins are inhibitors of differentiation, they may be playing this role by suppressing the 5S-A ASCL1-mediated pro-differentiation activity, thus effectively leaving cells locked in a non-proliferative immature neuronal-like state.

**Figure 4:**
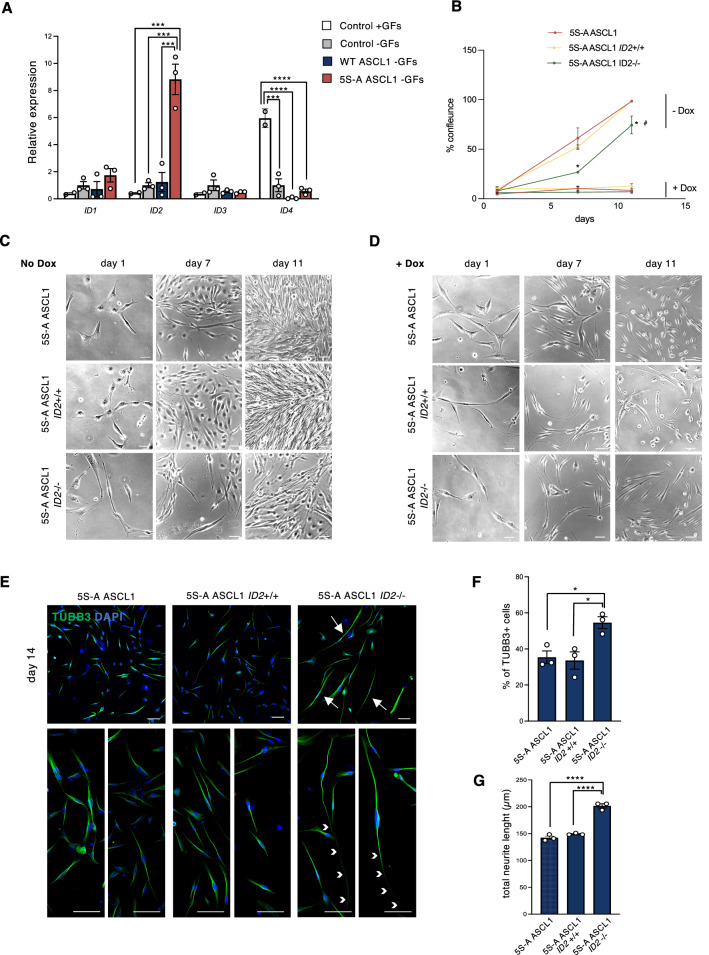
ID2 restrains differentiation induced by dephosphorylated ASCL1. (**A**) Relative mRNA expression of different members of the *ID* family at 7 days after WT and 5S-A ASCL1 expression. Data: mean ± SEM, normalized to *TBP*. n = 3 independent experiments; one-way ANOVA followed by the Bonferroni post-hoc test; ***p < 0.001; **** *p* < 0.0001. (**B**) Quantification of cell confluence. Each data point is mean ± SEM n = 3 independent experiments; one-way ANOVA followed by the Bonferroni post-hoc test; *p ≤ 0.05 between *ID2*^−/−^ and 5SA-ASCL1; #*p* ≤ 0.05 between *ID2*^−/−^ and *ID2*^+/+^. (**C**,**D**) Representative images of unedited control cells, *ID2* wild type cells and *ID2* homozygous knock-out cells, without (**C**) or with (**D**) dox-induced ASCL1 expression at increasing time points, as labelled. Scale bars: 100 μm. (**E**) Immunostaining for neuronal marker TUBB3 (green) at 14 days of culture in differentiation conditions. Scale bars: 100 μm. (**F**) Quantification of the % of TUBB3^+^ cells over the total DAPI^+^ cells at day 14. Data: mean ± SEM n = 3 independent experiments; one-way ANOVA followed by the Bonferroni post-hoc test; *p < 0.05. (**H**) Quantification of the total neurite length per cell. Data: mean ± SEM n = 3 independent experiments; one-way ANOVA followed by the Bonferroni post-hoc test; ****p < 0.0001.

We first looked at the expression of the different members of the ID family in response to ASCL1 expression and found a ninefold up-regulation of *ID2*, but not of *ID1*, *ID3* or *ID4,* by 5S-A ASCL1, in comparison to uninfected controls and WT ASCL1-expressing cells (Fig. 4A). We also detected increased *ID2* expression as early as 24 h after 5S-A ASCL1 induction (Fig. S6A). Interestingly, we also found strong upregulation of *ID1* and to a lesser extent of *ID2* and *ID4* upon 5S-A ASCL1 expression in the G166 cell line, in comparison to both WT-ASCL1 expressing cells and control conditions (Fig. S6B), suggesting that the molecular control of 5S-A mediated differentiation might be conserved across cell lines.

ID2 plays an important role in promoting survival and aggressiveness of GSCs^45–49^ and high ID2 inhibits oligodendrocyte differentiation resulting in OPCs that are resistant to maturation^50^; however, ID2’s role in cells that are undergoing neuronal differentiation is less clear. We hypothesized that 5S-A ASCL1-mediated upregulation of *ID2* might counteract the higher expression and activity of 5S-A ASCL1, thus restraining GBM cell differentiation.

To test the role of *ID2* in restraining GBM cell differentiation, we deleted the *ID2* gene using CRISPR/Cas9 technology in G144 cells expressing inducible 5S-A ASCL1 (Fig. S6C,D). Homozygous *ID2* deletion in these cells resulted in decreased cell confluence, but no spontaneous differentiation (Fig. 4B–D and Fig. S6E), a phenotype that is consistent with the reported role for *ID2* in maintaining glioma cancer cell stemness^45,46,48,49^. Interestingly, when we induced differentiation by growth factor removal and by dox treatment to induce 5S-A ASCL1, we observed enhancement of the pro-differentiation effect of 5SA-ASCL1 when *ID2* was deleted, in comparison to 5S-A ASCL1 control cells (both unedited cells and control CRISPR-treated cells with no *ID2* deletion), as evidenced by an increase in the fraction of TUBB3 + cells (Fig. 4E,F). Moreover, we found that the TUBB3 + cells displayed a greater extent of morphological differentiation characterized by very elongated neuronal processes (Fig. 4E,G). Altogether, we have identified several roadblocks to GBM cell differentiation and show that, when addressed in combination, a more effective differentiation can be achieved.

## Discussion

**Figure 5:**
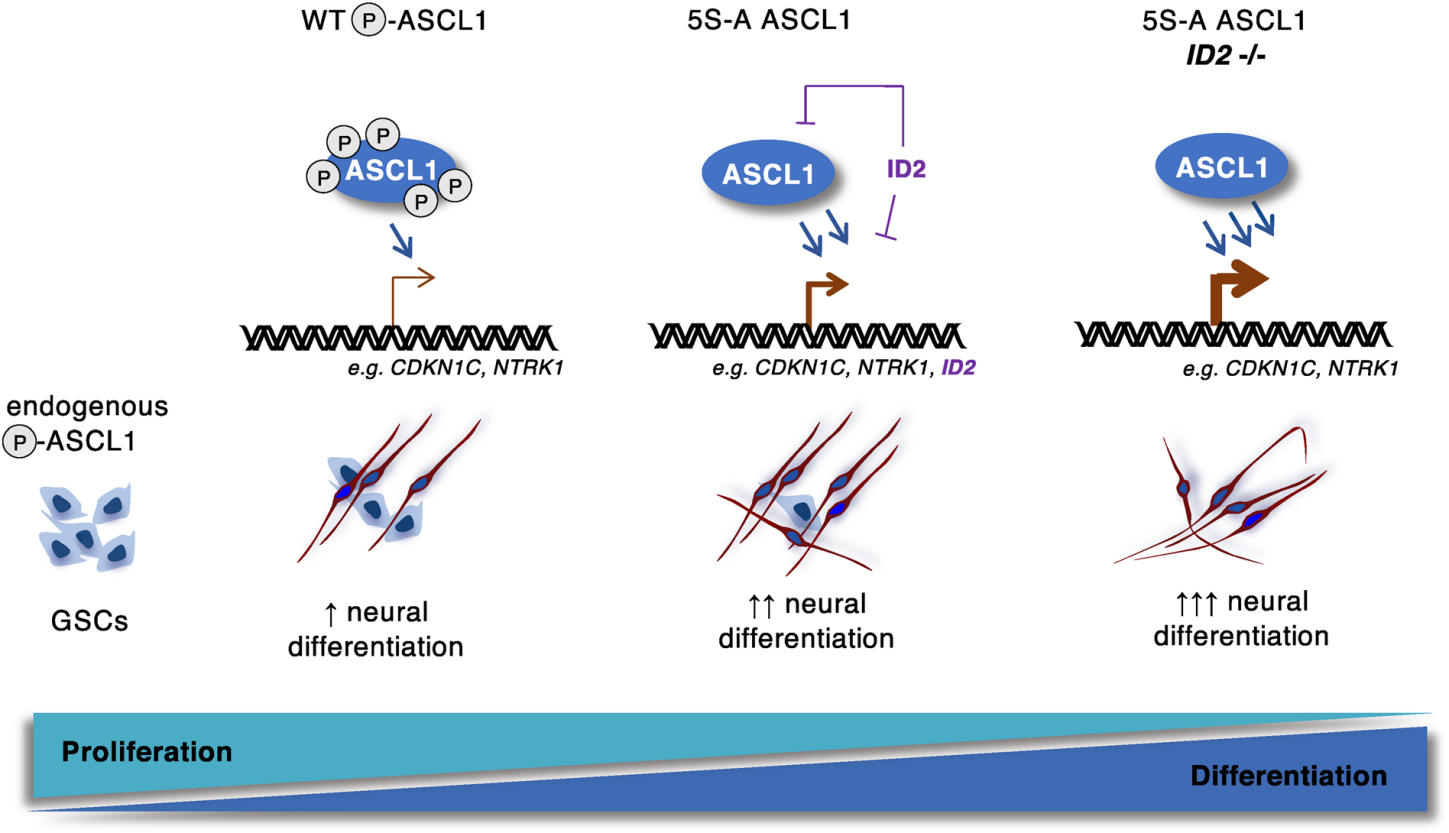
Model showing that ASCL1 dephosphorylation and reduction of *ID2* levels can enhance neuronal differentiation of glioblastoma stem cells.

In this study, we investigated the barriers to ASCL1-driven differentiation in GBM stem cells and showed that the oncogenic cell cycle environment maintains ASCL1 constantly phosphorylated, thus restraining its proneural activity. In contrast, preventing ASCL1 phosphorylation can drive a durable mitotic exit and promote differentiation. Previous work has shown that patients with GSCs expressing high ASCL1 might selectively benefit from directed differentiation therapy, as their GSCs were more responsive to Notch inhibitor-mediated differentiation^18^. It would thus be interesting to understand whether Notch inhibition affects ASCL1 phosphorylation. Notch inhibition is likely to result in increased ASCL1 levels^51,52^, and our data indicate that the rise in ASCL1 could trigger the activation of CDK inhibitors, such as CDKN1C, in turn slowing the cell cycle and limiting CDK-dependent phosphorylation. As cells start to differentiate with rising CDKN1C levels and decreasing CDK activity, increasing levels of dephosphorylated ASCL1 protein would enhance neurogenic activity, marking the transition to firm commitment to differentiation (Fig. 5).

Along with its established role in driving neuronal differentiation, ASCL1 is also essential for re-activation of quiescent stem cells in the adult brain^53–55^. Activity of un(der)phosphorylated ASCL1 in adult brain quiescent neural stem cells might either force differentiation or could alternatively re-activate the cell cycle to promote neuronal production. Similarly, the effect of phospho-mutant ASCL1 on quiescent GSCs in vivo might result in pushing them out of their quiescence and therapy- resistant state, making chemotherapies more effective^7^.

On ectopic expression of 5S-A ASCL1 in GBM cells, we see terminal differentiation was limited by concomitant upregulation of bHLH inhibitor ID proteins. We found that *ID2*, in particular, plays a key role in restraining phospho-mutant ASCL1 activity in GBM differentiation. Although the mechanism of ID2-mediated inhibition of phospho-mutant ASCL1 activity is still unclear, it is likely that both direct and indirect inhibition of key target genes mediates this antagonism^41,42^. Interestingly, the CDK inhibitor *CDKN1C*, which we found significantly upregulated by phospho-mutant ASCL1 (Fig. 1I), is also known to be repressed by ID2^56^. In both neural stem cells and neuroblastoma cells, ID2 competes with bHLHs and E proteins for *CDKN1C* activation^56^ and, in GBM, a gene signature with high *ID2* and low *CDKN1C* marks patients with a poorer prognosis^49^. Our data are consistent with the possibility that phospho-mutant ASCL1 activates key targets including *CDKN1C* to drive cell cycle exit and differentiation, but that concomitant activation of *ID2* counteracts elevated ASCL1 levels to ultimately limit expression of these ASCL1-driven pro-differentiation genes (model Fig. 5), so inhibiting terminal differentiation. Further support for our model comes from genome-wide analyses showing that *CDKN1C* and *ID2* are likely to be direct targets of ASCL1 in GBM and neuroblastoma cells^18,29^.

The mechanistic basis of the enhanced activity of 5S-A ASCL1 over WT ASCL1, for instance when comparing their ability to activate *ID2,* likely depends on greater chromatin binding by the phospho-mutant^29^. However, the reason for this enhanced binding is not well understood. One possibility is that ASCL1 might act as a homodimer to trans-activate a particular set of target genes, while 5S-A ASCL1 could preferentially form heterodimers with E-proteins to enhance binding to differentiation targets. Ascl1-E47 dimers promote neuronal differentiation more efficiently than Ascl1 alone in chicken spinal cord neural progenitor cells^57^, while the ability to homo versus heterodimerise has been shown for Neurog2 to be regulated by phosphorylation by GSK3-β^58^. It is also possible that phosphorylation influences the interaction of ASCL1 with other cofactors and chromatin remodelling complexes, thus impacting chromatin structure on a global genome scale, as well as changing affinity for DNA binding on specific target genes as shown in the paediatric cancer neuroblastoma^29^. Phosphorylation of ASCL1 could also affect its ability to concentrate in biomolecular condensates associated with high-level enhancer activation^59,60^.

Taken together, our findings lead to a model that suggests that phosphorylation of the developmental master-regulator ASCL1 plays a pivotal role in controlling differentiation of at least a subset of glioblastoma stem cells. It also points to new ways to reactivate the latent ability of GBMs to re-enter a post-mitotic neurogenic trajectory, a possible new therapeutic approach for this devastating disease.

## Methods

### Tumor samples and glioma stem cell culture

The glioblastoma stem cell (GSC) lines, G144 and G166, were provided as a kind gift from Prof. Steve Pollard, University of Edinburgh. The GSC lines, NCH644 and NCH421K, were purchased from the CLS Cell Lines Service GmbH. Cells were cultured as adherent monolayers (G144 and G166) or as neurospheres (NCH644 and NCH421K) in serum-free media (Sigma D8437) supplemented with 10 ng/mL mEGF (Peprotech), 10 ng/mL hFGF (R&D Systems), 1% v/v B27 (Invitrogen), 0.5% v/v N2 (Invitrogen) and 1 μg/mL of Laminin (Sigma) and incubated at 37 °C in 5% CO_2_.

### Differentiation assays

To study differentiation of GSCs, cells were plated on glass coverslips coated with ECM (Sigma E1270) 1:10 in PBS. The day after plating, medium was changed to medium without the growth factors EGF and FGF2 and with 1 μg/μL of dox to induce ASCL1 expression. Medium was replaced every 2–3 days. Cells were fixed at different time points and stained for differentiation markers. Quantification of TUBB3^+^ differentiated cells was performed using ImageJ software on at least 3 images coming from 3 independent experiments. Neurite quantification was performed using NeuronJ plugin in ImageJ software on 12 images coming from 3 different experiments.

### Proliferation assays

The confluence analysis of parental, WT and 5S-A ASCL1-expressing cells was performed using Incucyte (Zoom software), while the confluence analysis of *ID2*-CRISPR cells was done with ImageJ on images acquired with Nikon ECLIPSE TE2000-U. Confluence was calculated as the area covered by the cells over the total area of the image. Cell counts were performed by cell dissociation and automatic cell counts, using Countess (ThermoFisher), at the different time points. Percentage of cells incorporating EdU was performed using the Click-iT Plus EdU imaging kit (Life Technologies). Briefly, cells were treated with 10 μM of EdU for 24 h and were then fixed in 4% PFA and permeabilised. EdU was detected by adding the Click-iT Plus reaction cocktail and incubating for 30 min in the dark. Cells were then imaged using a Zeiss invert LSM510 confocal microscope.

### Generation of phospho-mutant ASCL1

Serine–Proline sites of the human ASCL1 cDNA were mutated into Alanine-Proline, using the QuickChange II Site-Directed Mutagenesis Kit (Stratagene) on pCS2-ASCL1. The mutated Serines are in position 93, 190, 194, 207 and 223. WT and 5S-A ASCL1 sequences were then cloned into the doxycycline-regulated pLVX-TRE3G vector (Clontech Takara) between BamH1 and MluI sites^28,29^.

### Lentiviral transduction

GBM cells were infected with a two-vector Tet-On lentiviral system (Clontech Takara). Lentiviral particles have been prepared as described before^61^. HEK 293 T cells were transfected using the ProFection kit (Promega, Calcium phosphate method) with the vectors of interest (pLVX-TRE3G containing either WT or 5S-A ASCL1 and the pLVX-Tet3G vector carrying the dox-inducible transactivator Tet-On 3G downstream of a CMV promoter) along with the packaging plasmids encoding for Rev, Gag-Pol, Tat and VSV-G. Two days after transfection, the cell supernatant containing the viral particles was harvested, filtered to remove debris and concentrated using Lenti-X Concentrator (Clontech Takara). After overnight incubation at 4 °C with the Concentrator, the supernatant was centrifuged at max speed for 1 h and the pellet was resuspended in 200μL of cell media. Lentiviral titre was determined using Lenti-X qRT-PCR Titration kit (Clontech Takara) on extracted viral RNA. For transduction, GBM cells were plated in a 24 well plate (2 × 10^4^ cells per well). The day after plating, cells were infected by replacing the media with 250 μL of media containing viral particles at multiplicity of infection (MOI) between 5 and 20 for the pLVX-TRE3G vectors and MOI 20 for the transactivator pLVX-Tet3G. Double selection of infected cells was performed with 1 μg/mL Puromycin and 500 μg/mL G418 and resistant cells were grown as pools. Induction of the transgene was achieved by dox treatment (1 μg/mL, Sigma).

### Immunocytochemistry

For immunostaining, cells were fixed in 4% PFA for 10 min at room temperature. Cells were permeabilized (0.3% Triton X100—PBS for 10 min); blocked for 45 min with 5% donkey serum in 0.01% Triton X100—PBS and incubated with the primary antibodies: rabbit anti-ASCL1 1:200 (Abcam, ab74065), mouse anti-TUBB3, 1:1000 (Covance MMS-435P), chicken anti-TUBB3, 1:1000 (Abcam ab41489) mouse anti-MAP2 1:200 (Sigma, M4403), rabbit anti-MKI67, 1:1000 (Abcam, ab15580). The secondary antibodies (1:800, Invitrogen) were incubated for two hours at room temperature. Cells were then stained with 1 μg/mL DAPI (Abcam ab228549) for 20 min at room temperature. Images were acquired with a confocal microscope (Zeiss invert LSM510 or SP5 Leica).

### Real-time PCR

Real-time PCR, qRT-PCR, was performed to detect mRNA levels of GBM cells upon WT and 5S-A ASCL1 induction. cDNA was synthesized using the QuantiTect Reverse Transcription Kit (Qiagen). PCR was performed using Quantifast SYBR Green PCR Kit (Qiagen). The thermal cycling conditions were: denaturation at 95 °C for 10 min and 50 cycles of 95 °C for 15 s and 65 °C for 1 min. Data is expressed as fold change in target gene expression relative to the housekeeping TBP gene, according to the 2^−ΔΔCT^ method^62^ and normalized to control levels. Experiments were performed in triplicates from 3 independent experiments. The list of primers is provided in Table S1.

### Immunoblotting

Cells were lysed on ice with RIPA buffer (Sigma) supplemented with 1X protease inhibitor cocktail (Roche). Proteins were separated on SDS-PAGE and immmunoblotted with rabbit anti-ASCL1 (1:1000, ab211327, Abcam), mouse anti-Rb (1:1000, 9309, Cell Signalling), rabbit anti CDKN1C (1:1000, 2557, Cell Signalling) and mouse anti-tubulin (1:1000, 66301, Proteintech). Secondary antibodies were horseradish peroxidase (HRP) conjugated (Amersham) and were detected with ECL Prime Western Blot Detection Kit (GE Healthcare).

### Phosphorylation assay

To detect phosphorylation of ASCL1, proteins were treated with 400 units of Lambda Protein Phosphatase (New England BioLabs) for 30 min at 30 °C. ASCL1 phosphorylation was visualised on 8% acrylamide gels polymerized with 20 μM “Phos-tag” (WAKO) and 40 μM MnCl_2_. After electrophoresis, “Phos-tag” gels were washed three times for 10 min with transfer buffer (14.4 g Glycine; 3 g Trizma Base; 800 mL H_2_O; 200 mL MeOH; final pH 8.3) plus 10 mM EDTA, and then once more with normal transfer buffer. Proteins were transferred to nitrocellulose membranes and immunoblotted with rabbit anti-ASCL1 (1:1000, ab211327, Abcam) and mouse anti-tubulin (1:1000, Sigma).

### Generation of ID2 KO cells with CRISPR/Cas9 technology

Cells were transfected using Amaxa 4D-Nucleofector transfection system (Primary Cell Optimization Kit). 6 × 10^5^ 5S-A ASCL1-expressing G144 cells were resuspended in P2 buffer, together with 20 µg of Cas9 Nuclease V3 (IDT, 1081058) and 8 µg of both synthetic chemically-modified sgRNA_1 and sgRNA_2 (Synthego; gRNA_1: CAGGTGTCCCCAAAACGAGG CCC; gRNA_2: GCACTGTGTGGCTGAATAAG CGG). Program DS-150 was used for nucleofection. After transfection, cells were cultured on laminin coated plates (laminin 5 µg/mL, Invitrogen, 3017-015) in GBM growth media, supplemented with Y-27632 (Stemcell Technologies, 72308). Clonal selection was performed by plating cells at clonal density in 60 cm^2^ dishes, three days after nucleofection. Clones were cultured for 12 days, manually picked and moved to a 96 well plate. To screen for *ID2* deletion, single clonal populations were firstly screened through PCR genotyping, using ID2_GF1 and ID2_GR1 primers (Table S1) and then confirmed by Sanger sequencing, using ID2_SeqF1 and ID2_SeqR1 primers (Table S1).

## Supplementary Information

Supplementary Information.
