# Supplementary material for: ASCL1 phosphorylation and ID2 upregulation are roadblocks to glioblastoma stem cell differentiation
Source: Sci Rep. 2022 Feb 11;12:2341. doi: 10.1038/s41598-022-06248-x (PMC8837758; doi:10.1038/s41598-022-06248-x)
Supplement: Supplementary file 1 — Supplementary Information. [file 41598_2022_6248_MOESM1_ESM.pdf]

Supplementary Information

ASCL1 phosphorylation and *ID2* upregulation are roadblocks to glioblastoma stem cell differentiation

Roberta Azzarelli[1,2,3,*], Aoibheann McNally[1,2], Claudia Dell'Amico[3], Marco Onorati[3], Benjamin Simons[1,4,5], Anna Philpott[1,2,*]

[1] Wellcome - Medical Research Council Cambridge Stem Cell Institute, University of Cambridge, UK; [2] Hutchison-MRC Centre, Department of Oncology, Hills Road, CB2 0XZ; [3] Department of Biology, Unit of Cell and Developmental Biology, University of Pisa, IT; [4] The Wellcome Trust/Cancer Research UK Gurdon Institute, University of Cambridge, UK; [5] Department of Mathematics, University of Cambridge.

* authors for correspondence (ra605@cam.ac.uk; ap113@cam.ac.uk)

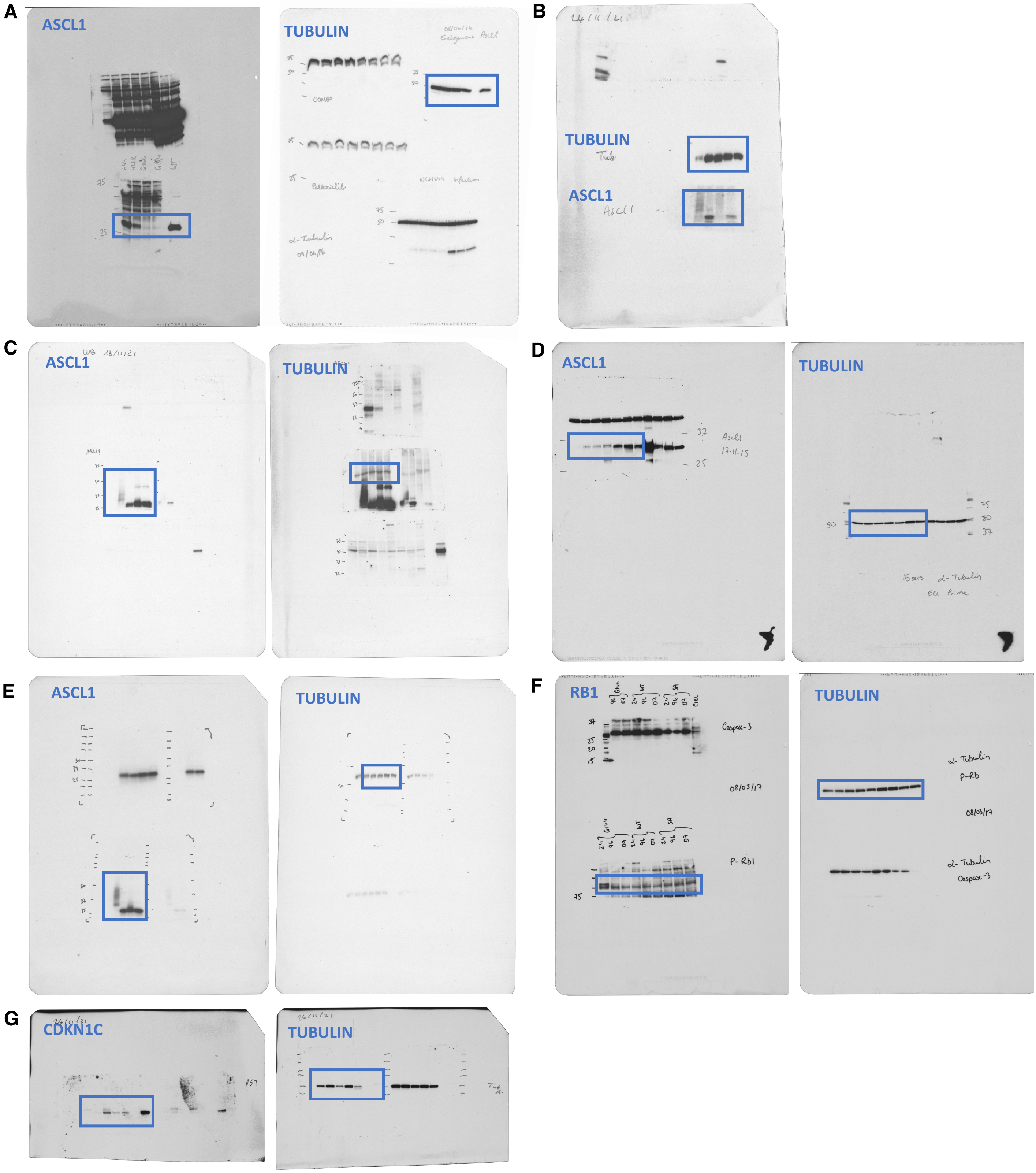

Figure S1. Full length Western Blot gels. Full length gels for Fig. 1A (A), Fig. 1B (B), Fig. 1C (C), Fig. S2F (D), Fig. S3B (E), Fig. S4E (F), Fig. S4C (G).

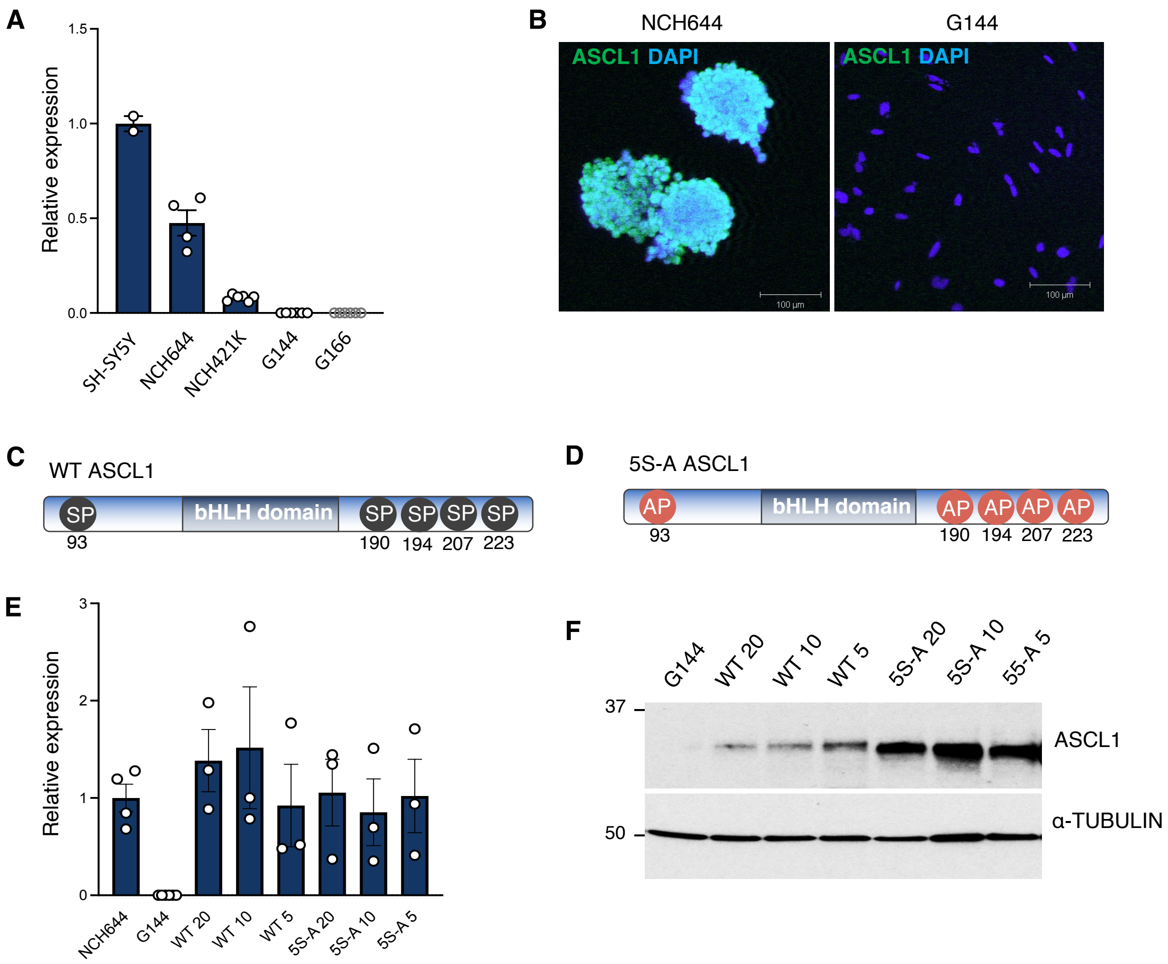

Figure S2. Expression of ASCL1 in GBM lines. (A) Expression of endogenous *ASCL1* mRNA in cell lines derived from primary human glioblastomas. The neuroblastoma line SH-SY5Y is used as a positive control. Data: mean ± s.e.m., normalized to *HPRT1*. (B) Immunofluorescence for ASCL1 in NCH644 and G144 cells. Scale bars: 100 µm. (C-D) Schematic representation of the 5 Serines, which are mutated to Alanine in phospho-mutant ASCL1 (5S-A ASCL1). (E-F) Expression of *ASCL1* mRNA (E) and protein (F) in G144 cells after 24 hours of dox induction. G144 cells have been transduced with dox inducible lentiviruses at different multiplicity of infection (MOI: 5, 10 or 20), as labelled. Data: mean ± s.e.m. normalized to *TBP*, n≥3

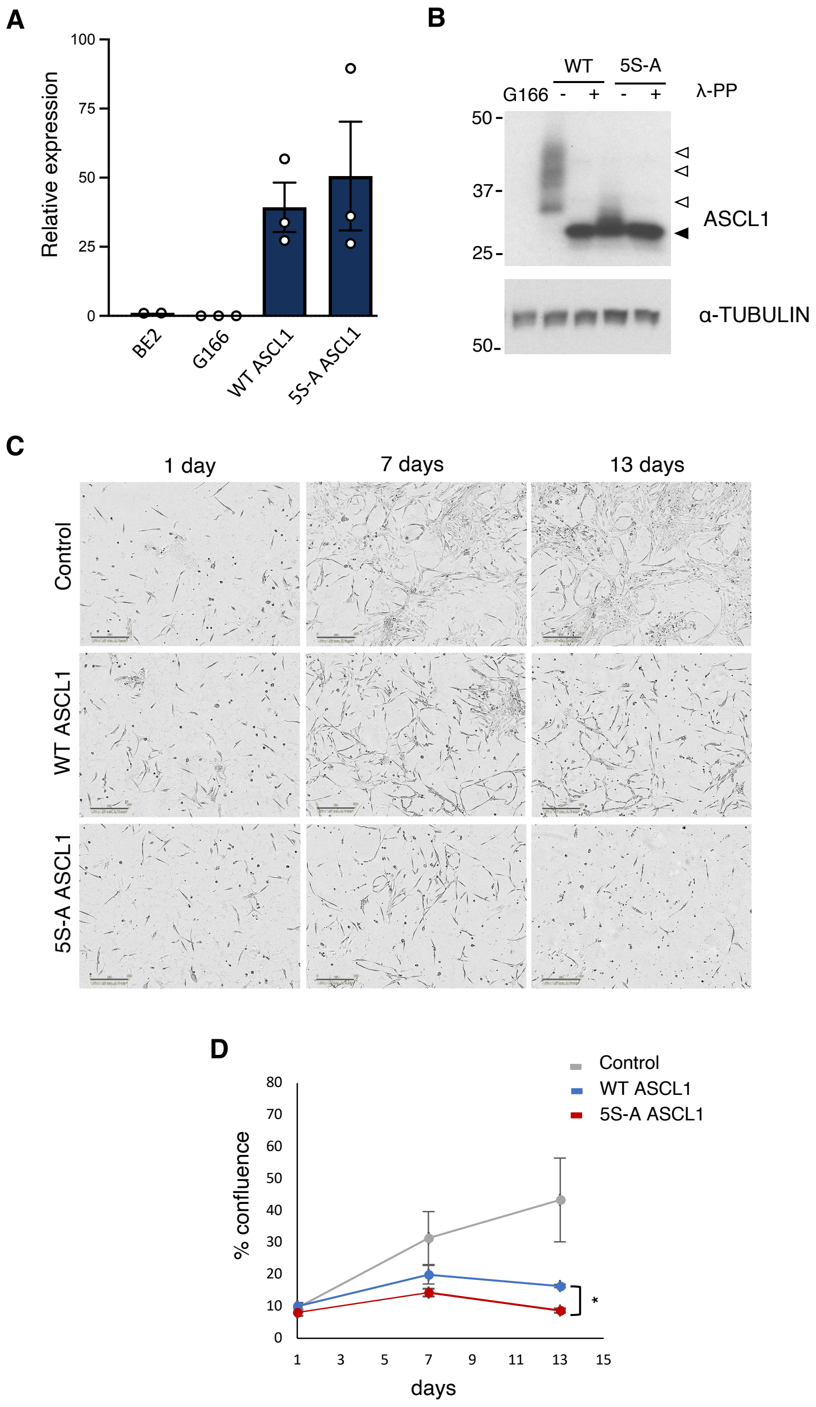

Figure S3. ASCL1 expression and phosphorylation in the G166 GBM cell line. (A-B) Expression of ASCL1 mRNA (A) and protein (B) in G166 cells 24 hours after WT and 5S-A ASCL1 induction, treated with and without phosphatase (λ-PP) as indicated. White and black arrowheads indicate phosphorylated and unphosphorylated ASCL1, respectively. (C) Representative images of G166 cells after growth factor withdrawal and dox induction of WT and 5S-A ASCL1 expression. Scale bars: 300 µm. (D) Quantification of cell confluence. Each data point is mean ± s.e.m. n=3 independent experiments; one-way ANOVA followed by the Bonferroni post-hoc test; *p≤0.05.

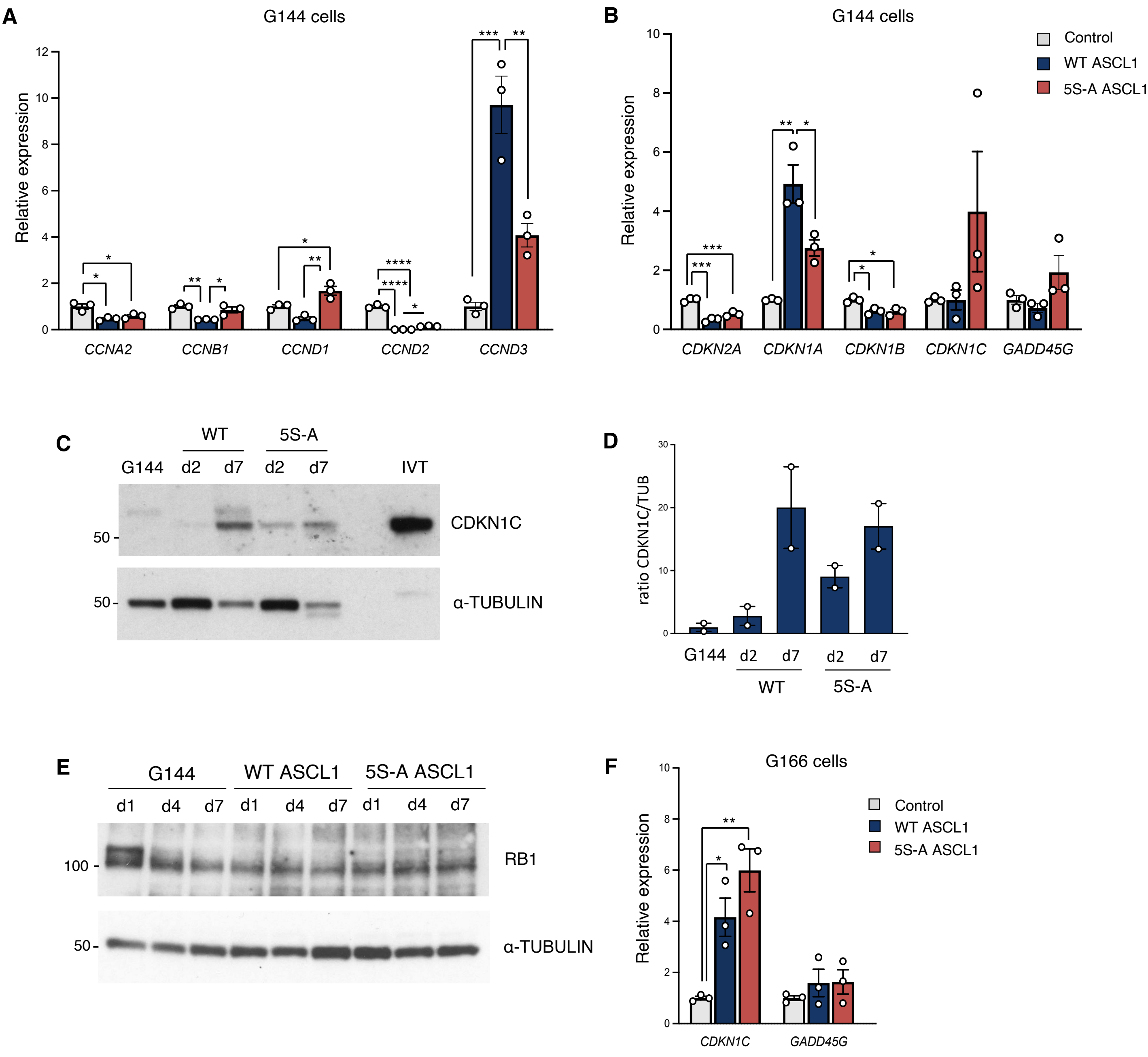

Figure S4. ASCL1-mediated regulation of cell cycle genes. (A-B) qPCR to measure expression of mRNAs for cyclins (A) and negative cell cycle regulators (B) in G144 cells, 24 hours post-WT or 5S-A ASCL1 induction. Data: mean ± s.e.m., normalized to *TBP;* n=3 independent experiments; one-way ANOVA followed by the Bonferroni post-hoc test; *p<0.05; ** p<0.01; ***p<0.001; ****p<0.0001. (C) Western blot of the cell cycle inhibitor CDKN1C in growing G144 cells and at day (d) 2 and 7 of growth factor withdrawal and dox induction of WT and 5S-A ASCL1 expression. (D) Quantification of the relative amount of CDKN1C, normalized to the level of TUBULIN expression. n=2 from different western blots from one experiment. *In vitro* translation (IVT) of CDKN1C has been used as a control. (E) Western blot showing expression and phosphorylation of the Retinoblastoma protein (RB1) in G144 cells at different days (d) of differentiation and upon induction of WT and 5S-A ASCL1 in differentiation conditions. (F) Expression of negative cell cycle regulators in G166 cells, 24 hours after WT or 5S-SA ASCL1 induction. Data: mean ± s.e.m., normalized to *TBP;* n=3 independent experiments; one-way ANOVA followed by the Bonferroni post-hoc test; *p<0.05; ** p<0.01.

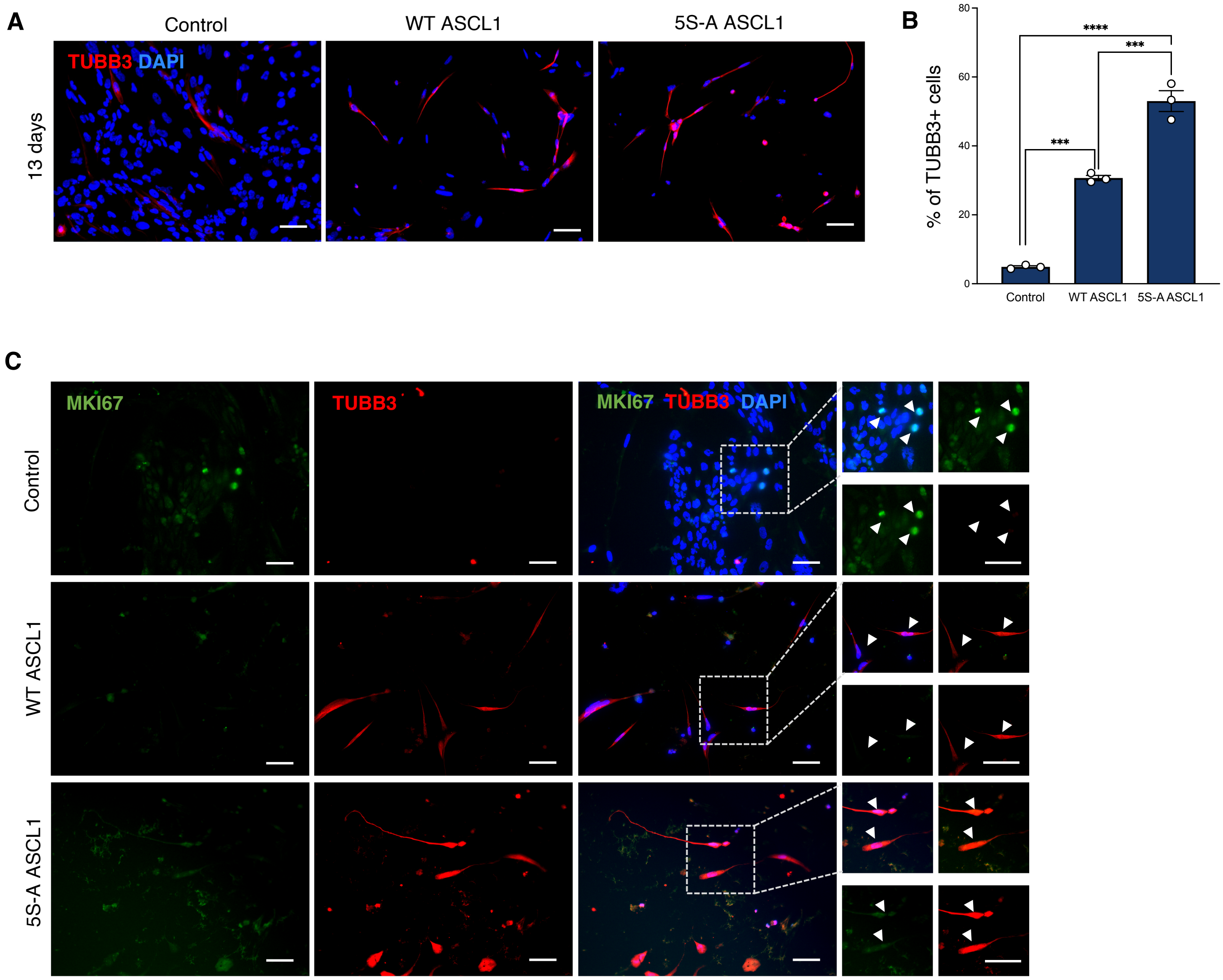

Figure S5. Effect of ASCL1 phosphorylation on G166 cell differentiation. (A) Immunostaining for the neuronal marker TUBB3 (red) in G166 cells cultured for 13 days without growth factors and in the presence of dox-induced WT or 5S-A ASCL1. Scale bars: 50 µm. (B) Quantification of the percentage of TUBB3^{+} cells over the total number of DAPI+ cells. Data: mean ± s.e.m. n=3 independent experiments; one-way ANOVA followed by the Bonferroni post-hoc test; ***$p<0.001$; ****$p<0.0001$. (C) Co-staining of TUBB3 and MKI67 at 13 days of differentiation upon WT and 5S-A ASCL1 induction. Scale bars: 50 µm.

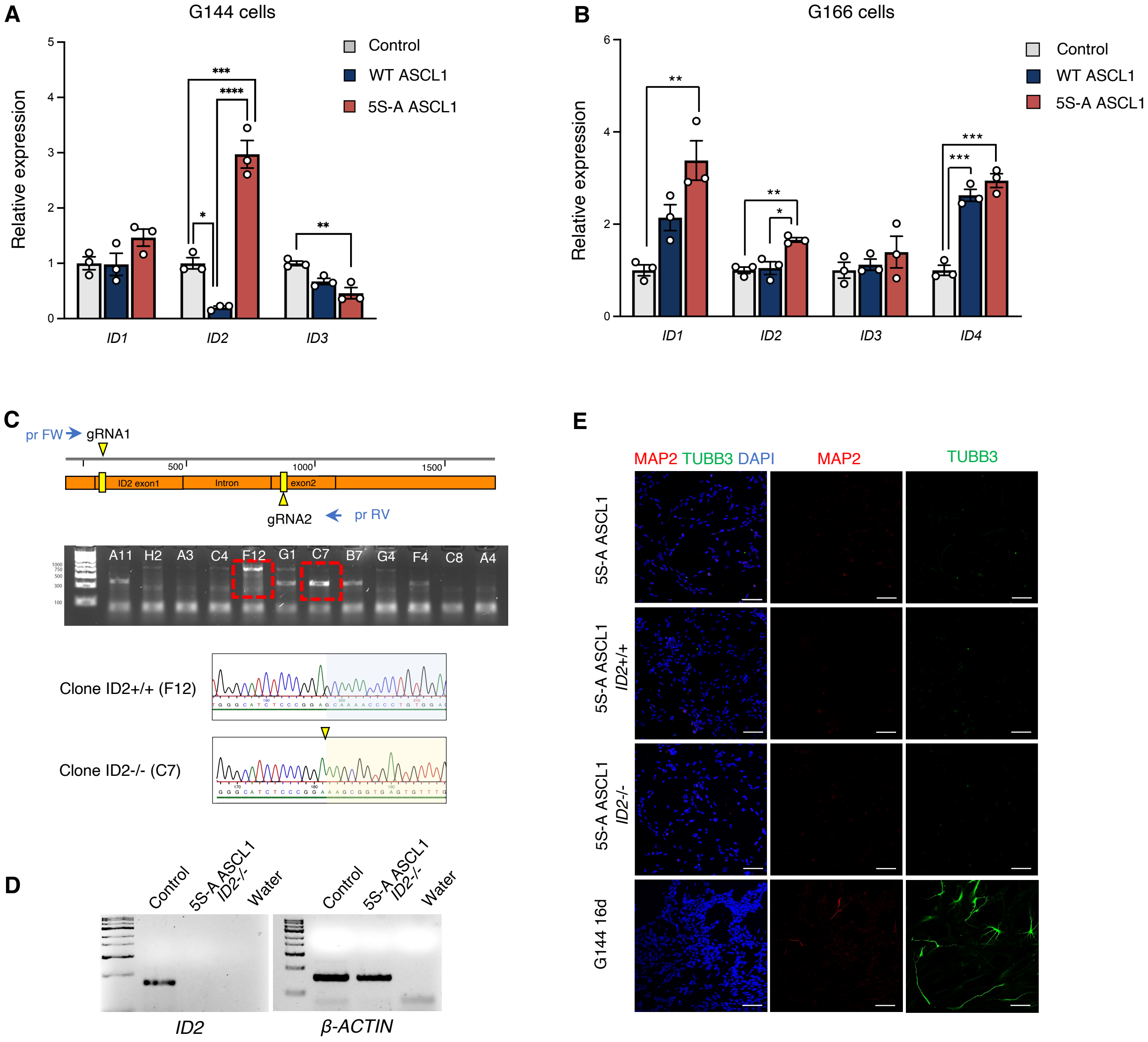

Figure S6. *ID* expression and generation of *ID2* knockout cells. (A-B) Relative mRNA expression of different members of the *ID* family, 24 hours after WT and 5S-A ASCL1 expression in G144 cells (A) and G166 cells (B).Data: mean ± s.e.m., normalized to *TBP*. n=3 independent experiments; one-way ANOVA followed by the Bonferroni post-hoc test; *p<0.05; ** p<0.01; ***p<0.001; ****p<0.0001. (C) Top panel: schematic diagram showing the position of the guide RNAs for *ID2* deletion; middle panel: PCR screening of different *ID2* mutated clones; bottom panels: snapshot of the sequencing data for *ID2*$^{+/+}$ F12 clone and *ID2*$^{-/-}$ C7 clone (yellow arrowhead indicates Cas9 cutting site). (D) PCR for *ID2* in Control and *ID2* CRISPR knockout cells. *β-ACTIN* is used as loading control. (E) Immunostaining for the neuronal markers TUBB3 (green) and MAP2 (red) in proliferative conditions. Positive control for the immunostaining: G144 cells after 16 days in differentiation conditions. Scale bar: 100 µm

	Forward Primer (5' – 3')	Reverse Primer (5' – 3')
**ASCL1**	CATCTCCCCCAACTACTCCA	AACGCCACTGACAAGAAAGC
**ASCL1 OE**	CTCAACTTCAGCGGCTTTGG	CTCATCTTCTTGTTGGCCGC
**PDGF**	GATACCTCGCCCATGTTCTG	CAGGCTGGTGTCCAAAGAAT
**PDGFRA**	CCACCGTCAAAGGAAAGAAG	CCAATTTGATGGATGGGACT
**MBP**	AAGAACTGCTCACTACGGCT	TGAATCCCTTGTGAGCCGAT
**SOX10**	GCTGAGTTGGACCAGTACCT	TCTGTCTTCACCTGGGCTTT
**ADRA2C**	GAGTACAACCTGAAGCGCACAC	GGAGGACAGGATGTACCAGGTC
**DBN1**	GAGGAAACTGAGGCAAAGAGGA	TCGGAGCCATCTTCATATGTGT
**NTRK1**	TTGCCTGCCTCTTCCTTTCTAC	ATTGTGGGTTCTCGATGATGTG
**OLIG2**	CAGAAGCGCTGATGG	TCGGCAGTTTTGGGT
**ID1**	ATCAGGGACCTTCAGTTGGAGC	GGAGACCCACAGAGCACGTAAT
**ID2**	CGACCCGATGAGCCTGCTAT	TCCGTGTTGAGGGTGGTCAG
**ID3**	GGTCACTGTAGCGGGACTTCTT	GTGGTTCATGTCGTCCAGCAAG
**ID4**	GCTCACTGCGCTCAACACCG	CTGGCTCGGGCTCAGGCGGC
**CCNA2**	GCACTCTACACAGTCACGGG	GTGTCTCTGGTGGGTTGAGG
**CCNB1**	TGTTGGTTTCTGCTGGGTGT	TGCCATGTTGATCTTCGCCT
**CCND1**	TTTGTTGTGTGTGCAGGGAG	TTTCTTCTTGACTGGCACGC
**CCND2**	CCGCAGTGCTCCTACTTCAA	GCCAAGAAACGGTCCAGGTA
**CCND3**	TTTGGGGCAGCACTGGTTTA	AGGCCAGGAAATCATGTGCA
**CDKN2A**	AGGGGTGCCACATTCGCTAA	GCCAGCCCCTCCTCTTTCTT
**CDKN1A**	GCACTTTGATTAGCAGCGGA	AGCCGAGAGAAAACAGTCCA
**CDKN1B**	AGAGACATGGAAGAGGCGAG	CCAAATGCGTGTCCTCAGAG
**CDKN1C**	GAGCCAATTTAGAGCCCAAAGA	AAGCTTTACACCTTGGGACCAG
**GADD45G**	GACACAGTTCCGGAAAGCACAG	AGCGTAAAATGGATCTGCAGCG
**TBP1**	AGCAGCAGCAACAGGCAGTG	TGGGGGAGGGATACAGTGGA
**β-ACTIN**	GCCCATCTACGAGGGGTATG	GTGGCCATCTCTTGCTCGAAG
**ID2_GF1**	CCGCCGAGTGCGGATAAAAG	
**ID2_GR1**	GTACGGGTAACACCGCGAAG	
**ID2_SeqF1**	AGCCGCCCCGCCGGGCTCGG	
**ID2_SeqR1**	GTAAGCATGCATTTACCAAA	

Table S1. List of primers for qPCR and ID2 sequencing
